# Contact lenses with non‑refractive opaque features for paediatric myopia: a randomised, safety pilot trial

**DOI:** 10.1038/s41433-026-04469-2

**Published:** 2026-04-22

**Authors:** Ravi C. Bakaraju, Jennie Diec, Daniel Tilia, Klaus Ehrmann, Ranjay Chakraborty

**Affiliations:** 1 nthalmic pty ltd, Sydney, NSW Australia; 2 School of Optometry and Vision Science, UNSW, Sydney, NSW Australia; 3 Myopia and Visual Development Lab, Caring Futures Institute, College of Nursing and Health Sciences, Flinders University, Adelaide, SA Australia

**Keywords:** Refractive errors, Paediatrics

## Introduction

Most optical myopia-control strategies employ competing defocus, extended depth of focus, or light scatter to modulate ocular growth [1]. A daily disposable, single-vision soft contact lens incorporating non-refractive, light-absorbing, opaque features was developed to alter the spatiotemporal retinal luminance profile during natural eye movement without inducing defocus or blur [2]. Computational modelling [2] indicates such features generate distinct retinal ganglion cell firing patterns compared with conventional designs, and visual performance in adults was subjectively superior to a dual-focus lens [3]. In-vivo relevance for myopia control remains unknown. Experimental evidence raises concern that partial occlusion could potentiate myopiagenesis [4], underpinning the need for paediatric safety data before larger efficacy trials. This 14-month, three-phase, within-participant crossover pilot in myopic children assesses safety, feasibility, and explores axial-length signals with the proposed non-refractive lens design.

## Methods

The study was conducted at two Australian sites with independent ethics approval and trial registration. Children aged 7–15 years with myopia suitable for contact-lens wear were screened; ocular pathology and prior myopia-control therapy were exclusion criteria. Seven children enrolled; six were dispensed with lenses and completed the study (see Supplementary Fig. 1). The test lens was a daily disposable Oculfilcon D soft lens matched to the control in diameter, base curve, refractive index, oxygen permeability, centre thickness, and edge design. Both provided single-vision correction; the test lens incorporated 28 rectangular (~350 × 100 μm) opaque features within the central 5 mm optic zone [3]. At dispensing (Phase 1, 0–6 months), participants were randomised to test lens in one eye and matched control in the fellow eye; assignments crossed over in Phase 2 (6–12 months), followed by bilateral control-lens wear in Phase 3 (12–14 months). Visits occurred at 2-month intervals. The primary variable was non-cycloplegic axial length measured by non-contact optical biometry. Safety assessments included slit-lamp examination, high-contrast visual acuity (HCVA), and participant-reported comfort. Analyses is descriptive with no inferential statistics.

## Results

**Fig. 1:**
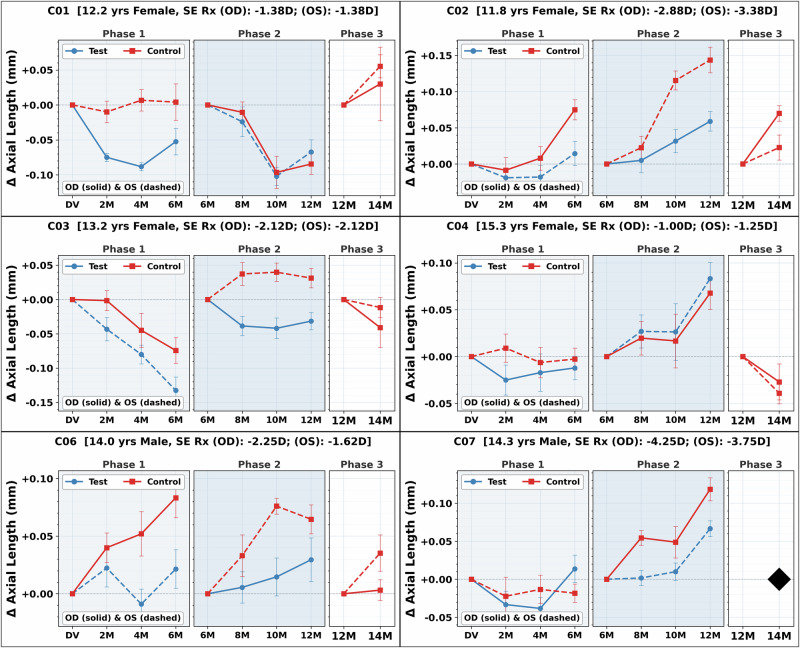
Eye-specific axial-length trajectories across the 14-month study period. Individual change in axial length (Δ mm) from baseline is shown for six participants (C01-C04, C06, C07; C05 withdrew before dispensing). Panel titles report participant ID, age, sex, and baseline spherical equivalent (OD/OS). Y-axis: Δ axial length (mm) from dispensing visit (DV, time zero) measured by non-contact optical biometry at 2-month intervals; all traces originate at zero. X-axis: DV to 14 months. Shaded regions denote Phase 1 (DV-6M), Phase 2 (6M-12M), and Phase 3 (12M-14M); lens assignments crossed over at 6 months, and both eyes wore control lenses after 12 months. Blue filled circles = test-assigned eye; red filled squares = control-assigned eye; colour follows lens type across crossover. Solid lines = OD; dashed lines = OS. Vertical bars = SD of repeated measures. Filled black diamond (C07, Phase 3) = missing month-14 value; no imputation performed. During Phase 3, axial-length trajectories were generally stable with no obvious short-term rebound following cessation of test lens wear.

Baseline characteristics, HCVA, adherence, and protocol deviations are summarised in Table 1; eye-specific axial-length trajectories are plotted in Fig. 1. Slit-lamp findings were unremarkable at all visits, with no corneal infiltrative events or vision-threatening adverse events. HCVA remained stable; no participant required a change in refractive correction. Comfort was generally acceptable. Axial-length trajectories were heterogeneous: in four participants, test-assigned eyes showed attenuated axial elongation during Phase 1 relative to fellow control eyes, with directional changes after crossover in Phase 2; in the remaining two, trajectories were similar or showed no clear attenuation. No group-level treatment effect is inferred; data illustrates within-participant patterns under real-world adherence to lens-wear.

**Table 1:** Baseline characteristics and study course for each participant, including presenting subjective refractive error, age at baseline, sex, ethnicity, parental myopia, high-contrast visual acuity (HCVA) across study visits, and average weekly lens wear.

Participant ID	Subjective refraction	Age at BL (years)/Sex	Ethnicity	Parental myopia	HCVA range (logMAR) worst to best		Average hours/week	
					Test	Control	Contralateral wear (test/control) for 12 months	Bilateral wear (control/control) for 2 months
C01	OD: −1.25/−0.25 × 155 OS: −1.25/−0.25 × 045	12.3/F	Caucasian	Both	0.14 to 0.00	0.14 to 0.00	77.5	84.0
*Experienced a 3 week non-wear period due to non-trial related hospitalisation and systemic treatment (Aug to Sep 2024), resulting in the 8 M visit being scheduled* ~ *3 months after 6 M visit*								
C02	OD: −2.75/−0.25 × 065 OS: −3.00/−0.75 × 115	11.8/F	Indian	Mother	0.00 to −0.10	0.00 to −0.10	54.6	70.0
*Reported consistent wear with no protocol deviations*								
C03	OD: −2.00/−0.25×112 OS: −2.00/−-0.25 × 175	13.2/F	Indian	Father	0.02 to −0.12	0.00 to −0.20	54.4	72.0
*Reported a 2-week non-wear before the 4 M visit*								
C04	OD: −1.00SPH OS: −1.00/−0.50 × 078	15.3/F	Caucasian	Both	0.04 to −0.20	0.00 to −0.20	69.0	84.0
*Alternated between study lenses and habitual spectacles reported at the 2 M visit, started melatonin at 6 M, and reported a two-week non-wear before 8 M due to depletion of test lenses, despite adequate supply been given, raising the possibility of deliberate swapping and potential use of test lenses in both eyes*								
C06	OD: −2.00/−0.50 × 075 OS: −1.25/−0.75 × 044	15.2/M	Caucasian	Mother	0.00 to −0.10	0.00 to −0.10	96.5	84.0
*Reported high adherence and melatonin use for sleep throughout the trial period*								
C07	OD: −4.00/−0.50 × 110 OS: −3.50/−0.50 × 050	14.0/M	Indian	Mother	−0.04 to −0.10	−0.02 to −0.10	54.6	No data
*Found to be wearing test lenses in both eyes at the 10 M visit. No objective data confirmed whether this occurred earlier in the study, raising concerns about protocol deviation and possible impact on study outcomes. C07 relocated overseas at 12 M*.								

Participant self-reported wear times in average days/week, hours/day.

Average hours/week was calculated by using the self-reported wear time, weeks between each visit and total weeks in lens wear.

The study protocol stipulated a minimum average weekly wear time of 30 hours.

C05 is not presented as they discontinued the trial following the dispensing visit and was lost-to-follow up.

C07 is missing Bilateral wear time data as they discontinued at the 12-month visit.

Six participants (4 female; baseline age 11.8–15.3 years) completed lens wear; one withdrew before dispensing (C05). Reported wear ranged from 4 to 7 days per week and ~5 to 15 h per day. Participant-specific events and protocol deviations, including periods of lens non-wear, medication changes, and adherence issues, are noted. Across 14 months, the test lens was worn safely with stable visual acuity, acceptable comfort, and no evidence of accelerated eye growth during wear or post cessation.

*OD* Right eye, *OS* Left eye, *BL* Baseline, *F* Female, *M* Male, *HCVA* High contrast visual acuity.

## Discussion and conclusion

The bilateral study design controls for age, season, ethnicity, parental myopia, baseline refractive error, and shared environmental factors within each child, isolating differences attributable to lens design. This design approach differs from defocus- or scatter-based myopia-control lenses by preserving single-vision correction and targeting temporal contrast through opaque features.

Axial-length signals were modest and heterogeneous. Crossover lens trials are vulnerable to misassignment when interocular prescriptions are similar; one participant was confirmed to have worn test lenses bilaterally at one visit, and undetected misassignments remain possible, potentially attenuating phase-specific differences. Non-wear periods may further dilute phase-linked contrasts. These observations highlight the need for rigorous lens-identity checks, improved wear-time capture, and prespecified per-protocol sensitivity analyses in larger trials.

Recent reports show that some defocus-based spectacle designs produce retinal shadows [5], raising the hypothesis that the proposed spatiotemporal contrast modulation may contribute to the mechanism behind their therapeutic effects rather than solely through static defocus. Testing this will require dedicated retinal imaging and electrophysiological studies.

Despite a small sample, protocol deviations, and non-cycloplegic measurements, this pilot study suggests that single vision lenses with opaque-feature do not exacerbate eye growth, supporting progression to larger randomised clinical trials.

## Supplementary information

Supplementary Figure 1 legend

## Data Availability

De-identified individual participant records for axial length and contact lens adherence, as well as the study protocol, will be made available upon reasonable request to the corresponding author. Access is subject to execution of a data confidentiality agreement with nthalmic Pty Ltd.
