# Supplementary material for: Contact lenses with non‑refractive opaque features for paediatric myopia: a randomised, safety pilot trial
Source: Eye (Lond). 2026 Apr 22;40(9):1412–5. doi: 10.1038/s41433-026-04469-2 (PMC13269692; doi:10.1038/s41433-026-04469-2)

***Supplementary Figure 1****: Flowchart showing the disposition of participants throughout the clinical trial, including the reasons for discontinuation.*


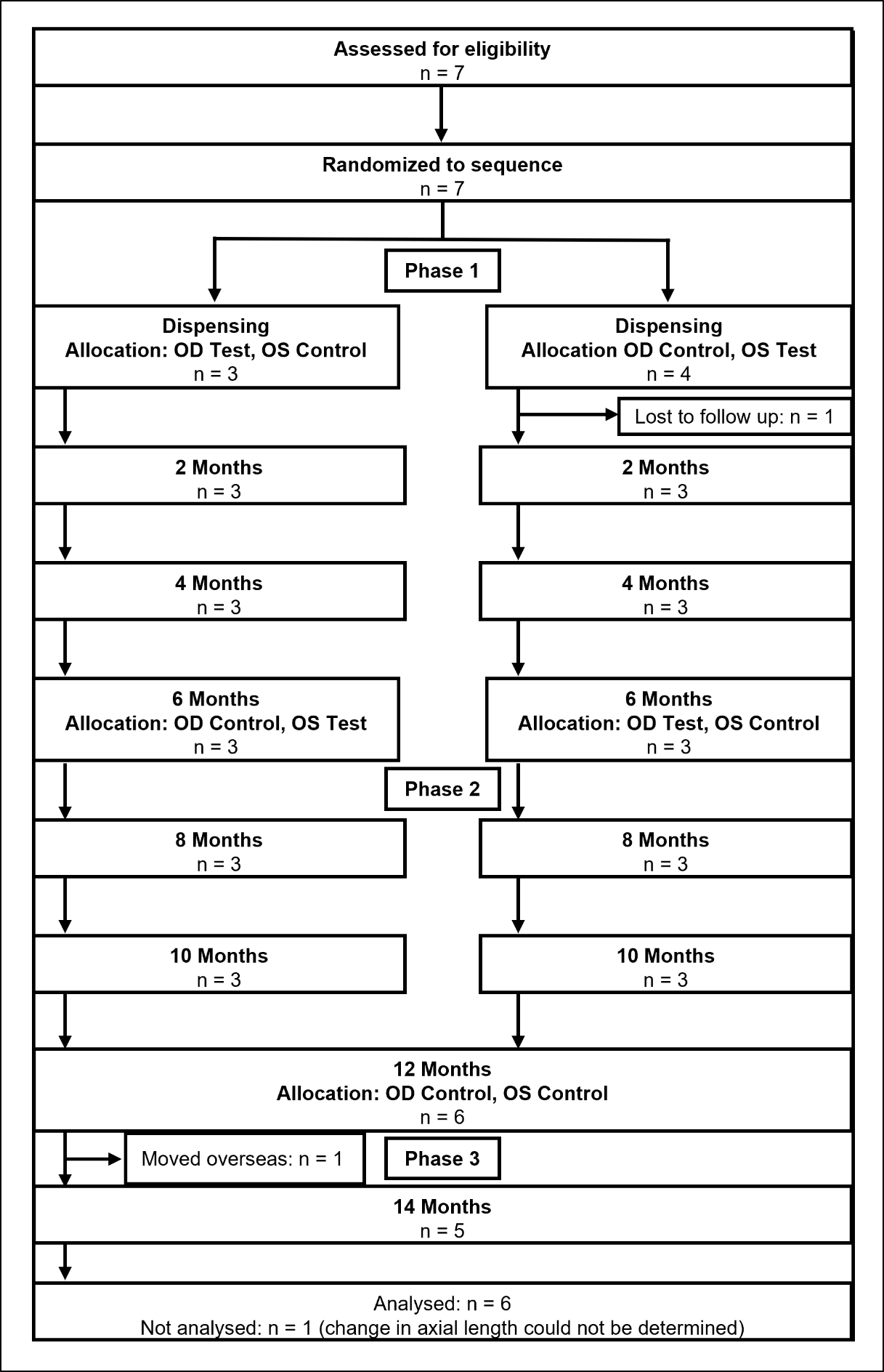

Supplement: Supplementary file 1 — Supplementary Figure 1 legend [file 41433_2026_4469_MOESM1_ESM.docx]
